# Study of therapeutic effect of different concentrations of imatinib on Balb/c model of cutaneous leishmaniasis

**DOI:** 10.3934/microbiol.2020010

**Published:** 2020-06-02

**Authors:** Mohsen Moslehi, Fatemeh Namdar, Mahsa Esmaeilifallah, Fariba Iraji, Bahareh Vakili, Fatemeh Sokhanvari, Seyed-Mohsen Hosseini, Faham Khamesipour, Zahra Sebghatollahi, Sayed-Hossein Hejazi

**Affiliations:** 1 Skin Diseases and Leishmaniasis Research Center, Isfahan University of Medical Sciences, Isfahan, Iran; 2 Department of Parasitology and Mycology, School of Medicine, Isfahan University of Medical Sciences, Isfahan; 3 Department of Dermatology, Isfahan University of Medical Sciences, Isfahan, Iran; 4 Department of Biostatistics & Epidemiology, School of Health, Isfahan University of Medical Sciences, Isfahan, Iran; 5 Sabzevar University of Medical Sciences, Sabzevar, Iran; 6 Shahid Beheshti University of Medical Sciences, Tehran, Iran

**Keywords:** imatinib, cutaneous leishmaniasis, therapeutic, Balb/c

## Abstract

Leishmaniasis, as a tropical and subtropical disease, is endemic in more than 90 countries around the world. Today, cutaneous leishmaniasis (CL) that affects more than 1.5 million people per year lacks a definitive treatment approach. Imatinib is an anticancer drug that inhibits the abnormal function of Bcr-Abl due to its tyrosine kinase inhibitor, and that was the reason why the drug was tested for CL treatment because protein kinases are essential enzymes in the *Leishmania* genus. In this study, the *L. major* CL model of Balb/c mice was produced by injection of the cultured metacyclic form of parasite at the base of the tail. The possible recovery of CL ulcers and determination of the optimum dose of imatinib against *Leishmania* amastigotes were evaluated. A significant decrease was observed in mice treated with amphotericin B (positive control group) as well as imatinib 50 mg/kg compared to the unreceived drug, negative control group (P<0.05). This study could be promising in gaining insight into the potential of imatinib as an effective treatment approach against CL.

## 1. Introduction

Leishmaniasis is a vector-borne protozoan disease caused by an obligatory intra-macrophage parasite of the genus *Leishmania*, which threatens 350 million people in more than 90 countries [1],[2]. As the second important protozoal disease after malaria, it is transmitted by the bite of female *Phlebotomus* sandflies that has a broad range of clinical manifestations due to the various host-parasite interactions, the genetic makeup of the infected host and different species of the parasite [3],[4]. The three primary common forms are reported in addition to the intermediate forms of the syndrome, including cutaneous leishmaniasis (CL), mucocutaneous leishmaniasis (MCL), and visceral leishmaniasis (VL). *Leishmania* parasites as dixenic organisms have two separate morphologies in their hosts; promastigote forms (an elongated and flagellated parasite) in the invertebrate sand-fly host and the amastigote form (a small, and non-motile parasite) in invertebrate hosts [5]–[7]. During blood meal, the infected sand-fly deposits promastigotes in the dermis of mammalian hosts, including humans, which then phagocytosed by the resident macrophages. In phagocytes, the parasite transforms into amastigote form. According to WHO, the incidence of CL in high-burden countries in total are about 1–1.5 million cases per year. As mentioned at present, there is no definitive standard for the treatment of CL [8],[9]. Few choice drugs are available for CL treatment to include pentavalent antimony compounds, sodium stibogluconate (pentostam) and meglumine antimoniate (glucantime); however, none of them are efficient due to long course treatment and high toxicity as well as drug resistance reported among different species of *Leishmania* [10],[11]. Hence, achieving an appropriate chemical compound for the definitive treatment of CL is an emergency.

**Figure 1.**
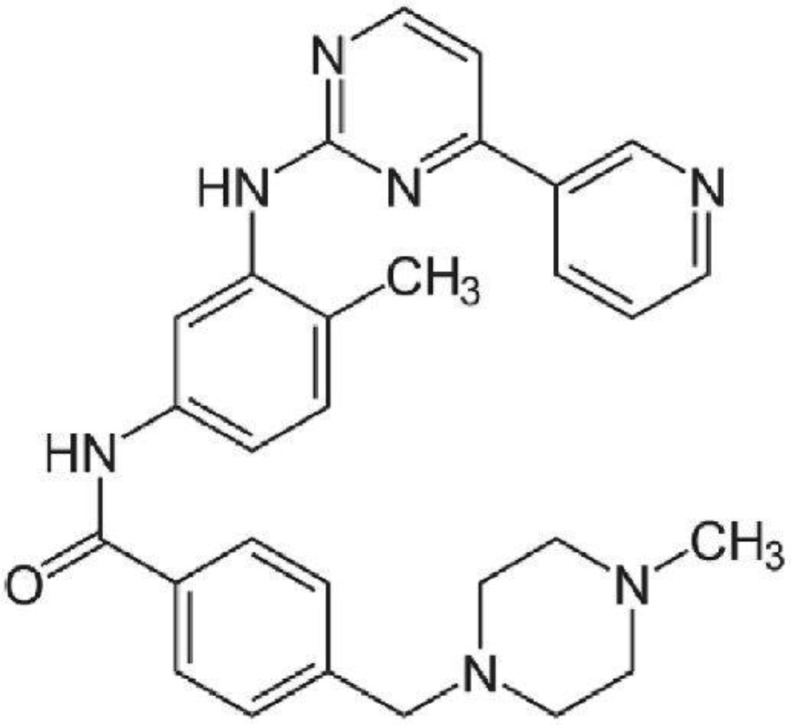
Chemical structure of Imatinib.

Imatinib is a small molecule kinase inhibitor (Figure 1). It is used in treating chronic myelogenous leukemia (CML), gastrointestinal stromal tumors (GISTs), and several other malignancies [12]. On the other hand, protein kinases are critical enzymes for the survival of *Leishmania* species [13],[14]. For instance, it is demonstrated a crucial role in macrophage invasion of parasite and could be an appropriate candidate for leishmaniasis therapy [15]. Also, Some of the imatinib physiochemical properties could provide us with the topical medication, for example, its log KP of -6.8 and Molecular weight of 493.6027 which are the most paramount ones for penetrating through the skin [16],[17].

After promising *in vitro* trial of the drug by our research team against the *Leishmania major* promastigotes [18], in the evolution of that study, the *in vivo* therapeutic effect of cream-based imatinib against *L. major* infection model of Balb/c mice at the doses of 50, 100 and 150 mg/kg has been evaluated in the present study.

## 2. Materials and methods

### 2.1. Leishmania parasites

To avoid genetic variations and unwanted mutations, *L. major* (MRHO/IR/75/ER) was routinely run on Balb/c mice at the Department of Parasitology & Mycology, School of Medicine, Isfahan University of Medical Sciences, Isfahan, Iran.

### 2.2. Ethical approval

The infected mice were kept in the animal houses under the condition of the Institutional Animal Care and Use Committee and Ethical Oversight Body and National Advisory Committee for Laboratory Animal Research, Iran, after the approval of the ethics committee under approval No: IR.mui.rec.1396.3.465.

### 2.3. Promastigote culture

The subiliac lymph nodes of a Balb/c mice previously infected with *L. major* were removed in a sterile condition. For initial isolation of the parasite after homogenization of exited lymph nodes in RPMI 1640 (Gibco, UK) cell culture medium, the antibiotics, penicillin 100 U/mL and streptomycin 100 µg/mL was added. The content was transferred to 25 mL flasks containing slanted blood agar medium (NNN medium) and incubated at 24 °C. The culture media were examined daily until the onset of proliferated promastigotes. After ensuring non-contamination, for mass production, the parasites were transferred to RPMI 1640 supplemented with 10% fetal bovine serum (FBS) (Invitrogen, Carlsbad, CA, USA), 100 µg/mL streptomycin, and 100 U/mL penicillin.

### 2.4. CL model of Balb/c mice

Fifty inbred Balb/c mice 4 to 6 weeks of age were purchased from Pasteur Institute of Tehran, Iran, and kept in a pathogen-free condition. All animal procedures were done in compliance with Animal Care, and the use of ethical committee approved protocols of Isfahan University of Medical Sciences. Each of the mice received subcutaneously at the base of the tail 1 × 10^6^ metacyclic forms of *L. major* (MRHO/IR/75/ER) promastigotes from the culture medium.

### 2.5. Preparation of imatinib

Imatinib (Gleevec) (Teva Pharmaceutical Industries) was purchased from Osveh Pharmaceutical Company, Tehran, I.R. Iran. The order of preparation of 3 different concentrations (50, 100, and 150 mg/kg) of it was given to the Department of Pharmacognosy, Faculty of Pharmacy, Isfahan University of Medical Sciences. Isfahan, I.R. Iran. The basis for determining these concentrations is the information obtained at the *in vitro* study [14].

### 2.6. In vivo assay

The infected Balb/c mice were divided into five groups, each ten (n = 10). All five groups received daily the prescribed drug for the group for three weeks. The groups were classified as follows: groups 1, 2, and 3 were treated with the 50,100 and 150 mg/kg imatinib, respectively. Group 4, the positive control, received intraperitoneally 4 mg/kg Amphotericin B and finally group 5, as negative control received cream daily without the effective drug (placebo). Measurements of ulcer sizes were done weekly immediately before starting treatment for the following four weeks using a digital colis vernier caliper. The size of the lesion was obtained by the average size of the two perpendicular diameters of the CL wound in the base of the tail.

### 2.7. Estimation of parasite burden

One week after the end of the treatment period, five mice were randomly selected from each group and sacrificed by the method of euthanasia. Spleens of them were removed and weighed. The parasite burdens were determined by the quantitative limiting dilution method. Briefly, spleens were entirely homogenized with a rotator homogenizer in 2 mL of complete Schneider's Drosophila Medium supplemented with 15% FBS and 0.1% gentamicin and were diluted with the same medium to a final concentration of 1 mg/mL. Fourfold serial dilutions of the homogenized spleens were then dispensed in the wells of a 96-well microliter plate and were cultured at 24 °C for three weeks. The presence or absence of motile parasites in each well was identified by direct observation under an inverted light microscope (40 ×). The parasite number in 1 mg of the tissue was calculated as follows: Parasite burden = -log _10_ (parasite dilution/tissue weight).

### 2.8. Statistical analysis

SPSS (version 22) was utilized for data analysis. For cutaneous infection, the means of the sizes of the lesions of treatment groups and controls were compared using the unpaired Student *t*-test. For parasite burden, the mean log_10_ values of parasites per gram of liver and spleen of treatment groups and controls were analyzed using the unpaired Student *t*-test. A *p*-value of <0.05 was considered statistically significant.

## 3. Results

### 3.1. Parasite culture

When amastigotes of *L. major* from infected mice were cultured *in vitro*, there was an exponential increase in cell number in the first 3–6 days, followed by a plateau in growth. The latter part of the growth curve was described as a stationary phase since there was no substantial increase in cell number, and these metacyclics are infectious forms for Balb/c mice.

### 3.2. CL model of Balb/c mice

After injection of 1 × 10^6^ metacyclic into each mouse, skin leishmanial nodules appeared at the site of injection (base of the tail) after 7 to 10 days. These lesions turned into ulcers after about three weeks.

### 3.3. Preparation of imatinib

Triple doses of the cream-based of imatinib, which were ordered to the Faculty of Pharmacy, were received in three different colors from the manufacturing department. These concentrations consisted of 50, 100, and 150 mg of imatinib cream-based, respectively.

### 3.4. In vivo assay

The mean of ulcer size measured at the last time in the negative (get a non-medicated cream, placebo) and the positive control groups (Amphotericin B) after three weeks of treatment was 6.98 ± 2.19 mm and 2.74 ± 0.5, respectively. Whereas, it was 3.59 ± 1.2 mm, 3.65 ± 0.5 mm, and 3.96 ± 0.5 mm in groups treated with the 50, 100, and 150 mg concentration of imatinib per ulcer, respectively (Table 1).

The ulcer size in mice treated with imatinib decreased significantly when compared to the untreated control group (*P* < 0.05). The highest decrease in ulcer size was observed in the amphotericin B treated group. A significant decrease was observed in mice treated with the amphotericin B as well as imatinib 50 mg per/kg compared to the unreceived drug, negative control group (*P* < 0.05).

**Table 1.** The mean of ulcer size in different test and control groups.

Group	Time			
	WO	W1	W2	W3
Imatinib 50 mg/kg	5.23 ± 1.3	5.23 ± 1.3	4.22 ± 1.1	3.59 ± 1.2
Imatinib 100 mg/kg	4.43 ± 0.5	4.24 ± 0.43	4 ± 0.57	3.65 ± 0.5
Imatinib 150 mg/kg	4.6 ± 0.5	4.42 ± 0.5	4.18 ± 0.6	3.96 ± 0.5
Amphotericin B 4 mg/kg	4.4 ± 0.6	4.04 ± 0.6	3.5 ± 0.6	2.74 ± 0.5
Negative Control	4.38± 1.49	5.56 ± 2.14	6.26 ± 2.18	6.98 ±2.19
P value	0.26	0.05	0.001	0.001

### 3.5. Estimation of parasite burden

**Figure 2.**
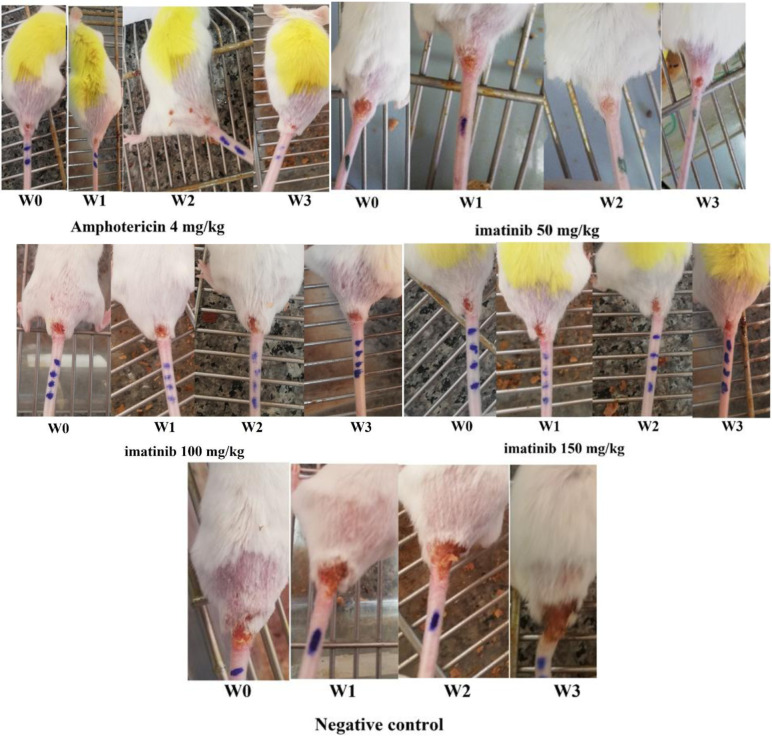
The ulcer size in different test and control groups.

The means of parasite burden in groups treated with the 50, 100, and 150 mg concentration of imatinib were 1.45, 2.42, and 2.37, respectively. It was 0.33 and 3.41 in the positive and negative control groups (Table 1). The parasite burden analysis revealed a significant decrease in the amphotericin B group compared with other groups. As for three groups of mice treated with imatinib, the highest decrease was observed in 50 mg concentration of the drug. From the parasite burden in groups of 100 and 150 mg of imatinib, there was no significant difference in the two groups (*P* > 0.05) (Figure 2) (Table 2).

**Table 2.** Estimation of parasite burden in different groups. Mice treated with imatinib 50 mg/kg illustrated the highest decrease compared to other concentrations (*P* < 0.05).

Spleen	N	Min	Max	Average	Sd	Sig
Imatinib 50 mg	5	1.36	1.51	1.45	0.08	* P < 0.05
Imatinib 100 mg	5	2.36	2.51	2.42	0.8	
Imatinib 150 mg	5	2.32	2.41	2.37	0.04	
Amphotericin 4 mg	5	0.29	0.38	0.33	0.04	
Negative control	5	2.30	4.52	3.41	0.1	

## 4. Discussion

Leishmaniasis is on the list of neglected diseases, and no vaccines or drugs to prevent infection are available. In terms of treatment of the disease, few drugs are available in the markets. On the other hand, as most types of the disease are zoonotic, there is no effective control strategy possible so, decisive and effective treatment is a method of prevention and control. However, none of the introduced drugs are efficient due to resistance issues, high cost, toxicity, a long period of treatment, and mainly the injectable use of these drugs [19],[20]. Hence, an effective protocol is needed to improve treatment strategies [21],[22]. Due to the limited variety of synthetic drugs effective in the treatment of CL, researchers have recently focused on the use of drugs that interfere effectively with the metabolic pathways and that inhibit parasite proliferation. Drugs that are already approved for the treatment of other diseases reduce the time and cost, which is needed for the identification of new drugs [23],[24]. Imatinib, as a tyrosine kinase inhibitor, is a synthetic chemical product which acts as a chemical agent for the treatment of CML and ALL [25]. It is found that tyrosine kinases are essential enzymes for *Leishmania* species. Researches revealed that imatinib is an effective drug in controlling metazoan parasites. For example, imatinib has a high schistosomicidal effect on *Schistosoma mansoni* *in vitro* [26],[27]. Some FDA-approved protein kinase inhibitors with anticancer effects also have shown anti-leishmanial effects. Imatinib, as an anti-parasitic drug, inhibits the growth and proliferation of *Leishmania* parasites by targeting *Leishmania* parasite tyrosine kinase. The family of protein kinases is the most important enzyme of *Leishmania* parasites to be involved in regulating cell cycle, control, differentiation, and response to stress during their complex life cycles [28]. For instance, it has a crucial role in macrophage invasion of parasite so, they could consider appropriate candidates to develop multi-drug therapy [29].

In the present study, due to the inhibitory effects of trypanosomatid protein kinase of imatinib, some *in vitro* and *in vivo* trials were designed by our research team to determine the killing effects of different concentrations of imatinib on *L. major*, that is, the main causative agent of CL in Iran [18].

The mean of ulcer size decreased significantly in mice treated with imatinib compared with the negative control group (*P* < 0.01). The highest decrease in ulcer size and parasite burden in imatinib treated mice was observed in 50 mg concentration. There was no significant difference in mice treated with 100 and 150 mg/kg of imatinib. In this study, the results showed that a concentration of 50 mg of imatinib is considered as an optimal concentration of the drug which could inhibit the development of CL ulcer which is quite similar to another protein kinase inhibitors such as sunitinib, lapatinib, and sorafenib with the highest efficiency on 50mg/kg dose [30].

Many studies have revealed that anticancer drugs have an inhibitory effect on the immune system of people who are taking these drugs, especially cellular immunity [31]–[33]. On the other hand, cellular immune responses possess a critical role against the *Leishmania* in involved cases [23]. Due to inhibition of the cellular immune response needed for anti-parasitic activities, it is possible that the two higher doses of imatinib (100 and 150 mg/kg) have not had a significant effect on disease control, while, a lower concentration of imatinib (50 mg/kg) significantly prevented the proliferation of parasites. Therefore, the optimal concentration of imatinib that can evoke a protective immunity against the parasites in the lower concentration.

In our previous study, the possible efficacy and determination of optimum concentrations of imatinib against proliferated promastigotes in the culture medium and amastigotes infected macrophage model were investigated. In that study, the anti-leishmanial effect of imatinib *in vitro* was proven; however, more investigations are needed to survey the efficacy of imatinib in the treatment and healing of CL wounds. It may be that using imatinib, together with other drugs, contributes to CL therapy [15]. On the other hand, altered physical forms of the drug, for example, imatinib-loaded magnetic nanoparticles, which is accompanied by nuclear magnetic nanoparticles imaging (MNP) that can facilitate drug delivery to the injured tissues and effectively incorporated into the CL treatment, just similar to what is used for targeted delivery [34].

## 5. Conclusions

Due to the anti-parasitic properties of imatinib, it could be a promising alternative drug for multidrug therapy against CL. However, further study is recommended to evaluate the possible efficacy of different physical forms of imatinib to have a more therapeutic effect on murine and human CL.

## References

[1] Ghorbani M, Farhoudi R (2018). *Leishmaniasis* in humans: drug or vaccine therapy?. Drug Des Devel Ther.

[2] Lindoso JA, Cunha MA, Queiroz IT (2016). Leishmaniasis–HIV coinfection: current challenges. HIV/AIDS.

[3] Bahrami F, Harandi AM, Rafati S (2018). Biomarkers of cutaneous leishmaniasis. Front Cell Infect Microbiol.

[4] Mendonça SC (2016). Differences in immune responses against *Leishmania* induced by infection and by immunization with killed parasite antigen: implications for vaccine discovery. Parasites Vectors.

[5] Apostolopoulos N, Mitropoulou A, Thom N (2018). Update on therapy and prevention of canine leishmaniasis. Tierarztl Prax Ausg K Kleintiere Heimtiere.

[6] Kaiming B, Yuyang C, Songnian Z (2018). Current visceral leishmaniasis research: a research review to inspire future study. BioMed Res Intl.

[7] Norouzinezhad F, Ghaffari F, Norouzinejad A (2016). Cutaneous leishmaniasis in Iran: Results from an epidemiological study in urban and rural provinces. Asian Pac J Trop Biomed.

[8] Adriaensen W, Dorlo TPC, Guido V (2018). Immunomodulatory therapy of visceral leishmaniasis in human immunodeficiency virus-coinfected patients. Front Immunol.

[9] de Vries HJ, Reedijk SH, Schallig HD (2015). Cutaneous leishmaniasis: recent developments in diagnosis and management. Am J Clin Dermatol.

[10] Alcântara LM, Ferreira TCS, Gadelha FR (2018). Challenges in drug discovery targeting TriTryp diseases with an emphasis on leishmaniasis. Int J Parasitol Drugs Drug Resist.

[11] Hotez PJ, Bottazzi ME, Strych U (2016). New vaccines for the world's poorest people. Annu Rev Med.

[12] Moen MD, McKeage K, Plosker GL (2007). Imatinib: a review of its use in chronic myeloid leukaemia. Drugs.

[13] O'Connell EM, Bennuru S, Steel C (2015). Targeting Filarial Abl-like Kinases: Orally Available, Food and Drug Administration–Approved Tyrosine Kinase Inhibitors Are Microfilaricidal and Macrofilaricidal. Int J Infect Dis.

[14] Kesely KR, Pantaleo A, Turrini FM (2016). Inhibition of an erythrocyte tyrosine kinase with imatinib prevents *Plasmodium falciparum* egress and terminates parasitemia. PloS one.

[15] Alvarez-Rueda N, Biron M, Le Pape (2009). Infectivity of *Leishmania mexicana* is associated with differential expression of protein kinase C-like triggered during a cell-cell contact. PLoS One.

[16] Hodgson J (2001). ADMET—turning chemicals into drugs. Nat Biotechnol.

[17] Potts RO, Guy RH (1992). Predicting skin permeability. Pharm Res.

[18] Moslehi M, Namdar F, Esmaeilifallah M (2019). Evaluation of different concentrations of imatinib on the viability of *Leishmania major*: An In Vitro study. Adv Biomed Res.

[19] Ponte-Sucre A, Gamarro F, Dujardin JC (2017). Drug resistance and treatment failure in leishmaniasis: A 21st century challenge. PLoS Negl Trop Dis.

[20] Vakili B, Eslami M, Hatam GR (2018). Immunoinformatics-aided design of a potential multi-epitope peptide vaccine against *Leishmania infantum*. Int J Biol Macromol.

[21] Scott P, Novais FO (2016). Cutaneous leishmaniasis: immune responses in protection and pathogenesis. Nat Rev Immunol.

[22] Vakili B, Nezafat N, Zare B (2020). A new multi-epitope peptide vaccine induces immune responses and protection against *Leishmania infantum* in BALB/c mice. Med Microbiol Immunol.

[23] Bekhit AA, El-Agroudy E, Helmy A (2018). *Leishmania* treatment and prevention: Natural and synthesized drugs. Eur J Med Chem.

[24] Vakili B, Nezafat N, Hatam GR (2018). *Proteome-scale identification of Leishmania infantum* for novel vaccine candidates: A hierarchical subtractive approach. Comput Biol Chem.

[25] Kennedy JA, Hobbs G (2018). Tyrosine kinase inhibitors in the treatment of chronic-phase CML: Strategies for frontline decision-making. Curr Hematol Malig Rep.

[26] Katz N, Couto FFB, Araújo N (2013). Imatinib activity on *Schistosoma mansoni*. Mem Inst Oswaldo Cruz.

[27] Beckmann S, Long T, Scheld C (2014). Serum albumin and α-1 acid glycoprotein impede the killing of *Schistosoma mansoni* by the tyrosine kinase inhibitor Imatinib. Int J Parasitol Drugs Drug Resist.

[28] Naula C, Parsons M, Mottram JC (2005). Protein kinases as drug targets in trypanosomes and *Leishmania*. Biochim Biophys Acta.

[29] Handman E, Bullen DV (2002). Interaction of *Leishmania* with the host macrophage. Trends Parasitol.

[30] Sanderson L, Yardley V, Croft SL (2014). Activity of anticancer protein kinase inhibitors against *Leishmania spp*. J Antimicrob Chemother.

[31] Matsushita M, Kawaguchi M (2018). Immunomodulatory effects of drugs for effective cancer immunotherapy. J Oncol.

[32] Hahn T, Polanczyk MJ, Borodovsky A (2013). Use of anticancer drugs, mitocans, to enhance the immune responses against tumors. Curr Pharm Biotechnol.

[33] Al-Abdely HM, Graybill JR, Bocanegra R (1998). Efficacies of KY62 against *Leishmania amazonensis* and *Leishmania donovani* in experimental murine cutaneous leishmaniasis and visceral leishmaniasis. Antimicrob Agents Chemother.

[34] Dahiya M, Dureja H (2016). Central composite designed imatinib-loaded magnetic nanoparticles. Recent Pat Nanotechnol.

